# Pathway-specific contribution of parvalbumin interneuron NMDARs to synaptic currents and thalamocortical feedforward inhibition

**DOI:** 10.1038/s41380-022-01747-9

**Published:** 2022-09-08

**Authors:** Eastman M. Lewis, Hayli E. Spence, Neha Akella, Andres Buonanno

**Affiliations:** Section on Molecular Neurobiology, Eunice Kennedy Shriver National Institute of Child Health and Human Development, National Institutes of Health, Porter Neuroscience Research Center, Bethesda, MD USA

**Keywords:** Neuroscience, Physiology, Schizophrenia

## Abstract

Prefrontal cortex (PFC) is a site of information convergence important for behaviors relevant to psychiatric disorders. Despite the importance of inhibitory GABAergic parvalbumin-expressing (PV+) interneurons to PFC circuit function and decades of interest in N-methyl-D-aspartate receptors (NMDARs) in these neurons, examples of defined circuit functions that depend on PV+ interneuron NMDARs have been elusive. Indeed, it remains controversial whether all PV+ interneurons contain functional NMDARs in adult PFC, which has major consequences for hypotheses of the pathogenesis of psychiatric disorders. Using a combination of fluorescent in situ hybridization, pathway-specific optogenetics, cell-type-specific gene ablation, and electrophysiological recordings from PV+ interneurons, here we resolve this controversy. We found that nearly 100% of PV+ interneurons in adult medial PFC (mPFC) express transcripts encoding GluN1 and GluN2B, and they have functional NMDARs. By optogenetically stimulating corticocortical and thalamocortical inputs to mPFC, we show that synaptic NMDAR contribution to PV+ interneuron EPSCs is pathway-specific, which likely explains earlier reports of PV+ interneurons without synaptic NMDAR currents. Lastly, we report a major contribution of NMDARs in PV+ interneurons to thalamus-mediated feedforward inhibition in adult mPFC circuits, suggesting molecular and circuit-based mechanisms for cognitive impairment under conditions of reduced NMDAR function. These findings represent an important conceptual advance that has major implications for hypotheses of the pathogenesis of psychiatric disorders.

## Introduction

The prefrontal cortex (PFC) is a site of convergence of long-range glutamatergic innervation from cortical and subcortical structures, including the thalamus. Convergent innervation allows the PFC to integrate multiple modalities of internally and externally generated information to produce goal-directed behaviors [1, 2] that are disrupted in psychiatric disorders, including Schizophrenia (Scz), bipolar disorder (BD), and autism spectrum disorder (ASD) [3, 4]. Inhibitory GABAergic parvalbumin-expressing (PV+) interneurons in PFC are implicated across psychiatric disorders [5–7], and PV+ interneurons exhibit electrophysiological and anatomical specializations that render them uniquely suited among GABAergic interneuron subtypes to coordinate neuronal activity [8, 9]. High-frequency PV+ interneuron-mediated inhibition temporally constrains pyramidal neuron excitation and generates gamma oscillations (≈30–100 Hz) that shape cortical function and are associated with information processing [10–17]. Patients diagnosed with Scz, BD, and ASD exhibit abnormal gamma oscillations as well as markers of altered PV+ interneuron metabolism and function [5–7, 14, 17–20]. Moreover, animal models that directly manipulate PV+ interneuron activity indicate that these fast-spiking interneurons contribute to behaviors relevant to psychiatric disorders, which depend on long-range innervation of the PFC [21–27]. Furthermore, PV+ interneuron-mediated thalamocortical feedforward inhibition (FFI) is altered in genetic models of Scz and ASD [28–30]. Despite the basic and translational significance of glutamatergic excitation of PV+ interneurons, controversy surrounding whether PV+ interneurons express functional N-methyl-D-aspartate receptors (NMDARs) in the adult PFC has persisted for over a decade [5, 31–36].

The hypothesis of NMDAR hypofunction in psychiatric disorders originates from the observation that acute administration of NMDAR antagonists produces a range of Scz-like symptoms in humans and is further supported by the discovery of NMDAR-encephalitis-induced psychosis [37–42]. More recently, there has been converging evidence that reduced NMDAR function in PV+ interneurons is relevant to the etiology of psychiatric disease. In adult humans and rodents, psychotomimetic NMDAR antagonists increase the baseline power of gamma oscillations, potentially as a result of selective inhibition of NMDARs on PV+ interneurons [43–47], but differences between acute NMDAR antagonism and Scz have been noted [48]. Moreover, selective deletion of the obligatory GluN1 subunit of the NMDAR by crossing GluN1^fl/fl^ mice with either PV-Cre or Ppp1r2-Cre mice, which targets a population of corticolimbic interneurons including PV+ interneurons, mimics NMDAR antagonist-induced increases in baseline gamma power and occludes the oscillatory and psychomotor effects of the non-competitive NMDAR antagonist MK-801 [49–53]; for review see [54]. Furthermore, NMDAR antagonism in adult mice reproduces molecular markers of altered PFC PV+ interneuron function observed in Scz [55], indicating an ongoing role for NMDAR in adult PV+ interneurons. Finally, the neuregulin/ErbB signaling pathway, which is genetically associated with psychiatric disease, downregulates NMDAR but not α-amino-3-hydroxy-5-methyl-4-isoxazolepropionic acid receptor (AMPAR) function in cortical and hippocampal PV+ interneurons [56, 57].

In conflict with these observations underscoring a physiologically important role of NMDARs in PV+ interneurons in vivo, previous slice electrophysiological studies using traditional electrical stimulation reported that synaptic NMDAR currents are either undetectable or extremely limited in adult mPFC PV+ interneurons [32, 33, 58], and that PV+ interneurons in juvenile animals exhibit smaller NMDAR currents than pyramidal neurons and other GABAergic interneurons (see ref. [59]). These observations, and the hypothesis that the long duration of NMDAR currents conflicts with temporal precision that is a hallmark of PV+ interneurons, have been interpreted to indicate that PV+ interneuron NMDARs have limited significance to mature PFC function [5, 34, 35]. Although this view is inconsistent with the large body of in vivo pharmacological and genetic literature discussed above, and a more recent study using optogenetic stimulation of long-range inputs to adult PFC fast-spiking interneurons [36], it may have persisted because the traditional approaches used to measure NMDAR currents in PV+ interneurons have limitations (see Discussion) and concrete examples of the impact of NMDAR activity in PV+ interneurons on adult PFC circuit function remain elusive. Therefore, resolving this issue is critical to understanding basic mechanisms of cortical circuit function, and for refining hypotheses of how NMDAR hypofunction contributes to cognitive deficits associated with psychiatric disorders. Utilizing a combination of experimental approaches not used to address this question previously, including fluorescent in situ hybridization, glutamate uncaging and pathway-specific optogenetics, we resolve this controversy by demonstrating that the vast majority of adult PV+ interneurons express functional NMDARs. Furthermore, we demonstrate that synaptic NMDAR contribution to PV+ interneuron EPSCs is pathway-specific and that NMDAR activity in PV+ interneurons is important for thalamo-prefrontal cortical FFI.

## Methods

(See Supplementary Information for details).

### Animal subjects

Adult (P70-100) male and female mice were used for all experiments (Supplementary Fig. 1). C57BL/6J mice were obtained from Jackson Laboratories (Bar Harbor, ME, USA) or bred in house for in situ hybridization. PV-Cre::TdTomato, PV-Cre::GluN1^fl/fl^, and Cre-negative::GluN1^fl/fl^ littermate controls were bred in house. Procedures were performed in accordance with NIH Animal Welfare guidelines.

### Fluorescent in situ hybridization

Fluorescent in situ hybridization for RNA coding for parvalbumin (*Pvalb*), GluN1 (*Grin1*), and GluN2B (*Grin2b*) experiments were performed on adult mouse brain sections (12 μm) using the RNAscope Fluorescent Multiplex Assay (Advanced Cell Diagnostics (ACD); Newark, CA, USA) as previously described [60]. Images were acquired using a Zeiss LSM 780 (Carl Zeiss Microscopy; White Plains, NY, USA) microscope with a 20x objective at 2x zoom. Image acquisition settings were kept constant.

### Electrophysiology

Whole-cell voltage-clamp recordings were made in coronal mPFC slices (300 µm). Adult PV-Cre::TdTomato mice were used to measure whole-cell and synaptic currents in PV+ interneurons and adult PV-Cre::GluN1^fl/fl^ mice and Cre-negative::GluN1^fl/fl^ littermate controls were used to measure FFI in pyramidal neurons. The experimenter was blind to genotype for recording and analysis of FFI.

#### Data acquisition

Recording pipettes were filled with Cs+-based internal solution. Recordings were acquired at 20 kHz and low-pass filtered at 10 kHz. Access resistance (Ra) was monitored for the duration of each experiment, and data was excluded when Ra changed >25% or exceeded 25 MΩ. Except for MK-801 (1 mM in the recording pipette [61] during recordings of FFI; Tocris; Minneapolis, MN, USA), drugs were bath-applied at the following concentrations: MNI-Glutamate (MNI-Glu; 50 µm; Tocris); 2,3-Dioxo-6-nitro-1,2,3,4-tetrahydrobenzo[*f*]quinoxaline-7-sulfonamide (NBQX; 10 µM; Tocris); picrotoxin (100 µM; Tocris); and tetrodotoxin (TTX; 1 µM; Tocris); 4-Aminopyridine (4-AP; 100 µM; Sigma Aldrich; St. Louis, MO, USA); D-AP5 (50 µM; Tocris); D-serine (100 µM; Tocris; glutamate uncaging). Recording artificial cerebrospinal fluid (aCSF) contained 1.3 mM Mg^2+^ and 2.5 mM Ca^2+^. To facilitate Chronos-mediated neurotransmitter release with TTX and 4-AP [62] (PV+ interneuron EPSC recordings) aCSF was modified to contain 4 mM Ca^2+^ [63]. Ultraviolet (glutamate uncaging) and blue (optogenetic stimulation) illumination was generated using a CoolLED pE-300^ultra^ (CoolLED; Andover, UK) and delivered to the slice through a ×40 objective.

### Stereotaxic injection

Microinjections (0.6 µl) of AAV5-Syn-Chronos-GFP (Addgene; Watertown, MA, USA) were targeted to either contralateral mPFC or ipsilateral medial dorsal region of the thalamus in 4–5-week-old mice anesthetized with isoflurane.

### Data analysis

CellProfiler 4 (www.cellprofiler.org; Broad Institute; Cambridge, MA, USA) [64] was used for automated in situ hybridization analysis. Electrophysiological data was filtered offline at 2 kHz or 1 kHz (single-trial glutamate uncaging measurements) and analyzed using Clampfit (Molecular Devices; San Jose, CA, USA). Statistical analyses (two-tailed, *p* < 0.05 for significance) were conducted in GraphPad Prism (Version 8.4.3; GraphPad Software; San Deigo, CA, USA). The Wilcoxon signed ranks test (paired comparisons) or the Mann–Whitney test (unpaired comparisons) were used. *p*-values for glutamate uncaging LED-response relationships were obtained from a simple linear regression. Data represented as mean ± SEM and *p* < 0.05 was the significance threshold.

## Results

### PV+ interneurons throughout the adult mouse mPFC co-express NMDAR transcripts necessary to encode functional receptors

**Fig. 1:**
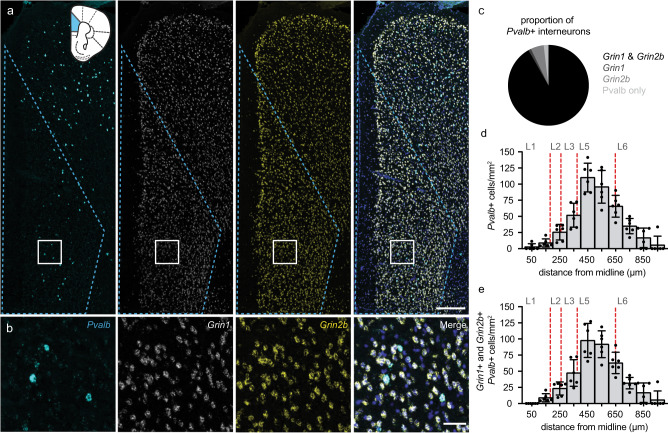
Adult mouse mPFC PV+ interneurons co-express NMDAR subunit transcripts that can encode functional NMDARs. **a** Representative images (20x with 2x zoom) of DAPI-stained (*blue; shown in merged images only*) mPFC sections showing in situ hybridization to *Pvalb* (*cyan*), *Grin1* (*white*) and *Grin2b* (*yellow*) transcripts. Dashed blue lines mark the region used for quantification, which is represented on a coronal diagram with PL and ILA in blue. Scale bar indicates 250 μm. **b** Zoomed images of the white inset in **a**. Scale bar indicates 50 μm. **c** Proportion of *Pvalb*+ interneurons containing *Grin1* and *Grin2b* RNA (black; 91.8% ± 2.4%), *Grin1* RNA only (dark gray; 1.1% ± 0.7%), *Grin2b* RNA only (medium gray; 5.2% ± 1.8%) or neither NMDAR RNA (light gray; 1.9% ± 0.9%) (333 *Pvalb*+ cells counted; *n* = 6 mice; 55.7 ± 5.8 *Pvalb*+ cells/mouse). **d**, **e** Density (cells/mm^2^) of *Pvalb*+ interneurons (**d**) and *Pvalb*+ interneurons containing both *Grin1* and *Grin2b* transcripts (**e**) across the depth of mPFC in 100 µm bins. Red dashed lines indicate the boundaries of cortical layers (L1–L6) defined according to Anastasiades et al.^68^. *Pvalb*+ cells expressing *Grin1* and *Grin2b* transcripts are distributed similarly to *Pvalb*+-labeled cells, which are densest in layers 5 and 6. Data are represented as mean ± SEM.

NMDARs are comprised of two obligatory GluN1 subunits and two GluN2 (or more rarely GluN3) subunits that assemble into functional heterotetrameric ionotropic ligand-gated channels [65, 66]. To analyze the proportion of PV+ interneurons in the prelimbic (PL) and infralimbic (ILA) adult mouse mPFC that co-express NMDAR subunits that may form functional receptors, we used a multi-fluorescent (3-fluorophores) in situ hybridization approach (RNAscope) with probes to identify RNAs encoding GluN1 (*Grin1*) and GluN2B (*Grin2b*) in parvalbumin (*Pvalb*) containing nuclei (Fig. 1a, b). *Grin2b* was chosen because it is abundantly and widely expressed in adult mouse cortex and, importantly, co-assembly of GluN2B with GluN1 is sufficient to form functional diheteromeric receptors. An automated image analysis pipeline [60] (see Methods and Supplementary Methods) was used to quantify the proportion of *Pvalb+* interneurons that co-express *Grin1* and *Grin2b* mRNAs in an unbiased manner (Fig. 1c). We found that the vast majority of *Pvalb+* interneurons (91.8 ± 2.4%) co-express *Grin1* and *Grin2b* transcripts, greatly outnumbering those only expressing either *Grin1* (1.1 ± 0.7%), *Grin2b* (5.2 ± 1.8%) or *Pvalb* (1.9 ± 0.9%; Fig. 1c). To examine the layer-dependent distribution of *Grin1/Grin2b/Pvalb-*expressing cells, we compared the density (cells/mm^2^) of all *Pvalb+* interneurons (Fig. 1d) to the density of triple-positive neurons (Fig. 1e) in 100-μm intervals across mPFC layers. Consistent with previous reports [67, 68], *Pvalb+* interneurons are present in all mPFC layers and are most concentrated in layers 5 and 6 (Fig. 1d). Furthermore, the distribution of *Pvalb+* interneurons expressing *Grin1* and *Grin2b* transcripts closely follows the general distribution of all *Pvalb+* interneurons (Fig. 1d, e). Therefore, virtually all mPFC PV+ interneurons co-express NMDAR subunit transcripts required to produce functional NMDARs, regardless of laminar location.

### Electrophysiological identification of NMDAR-mediated currents in adult PV+ interneurons

**Fig. 2:**
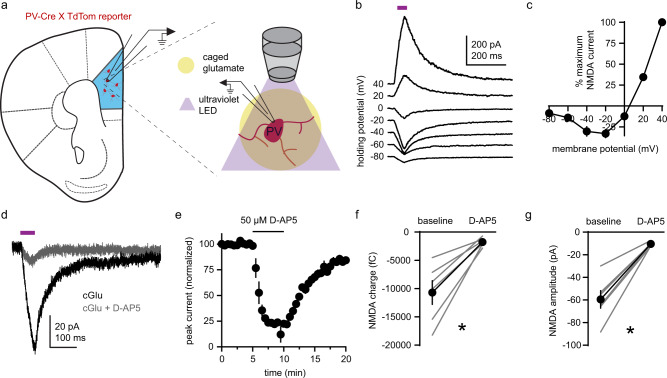
Electrophysiological identification of NMDAR-mediated currents in adult PV+ interneurons. **a** Schematic of experimental design. Whole-cell glutamatergic currents (_UV_EPSCs) were evoked by uncaging glutamate (MNI-Glu; 50 µM) with an ultraviolet light pulse in the presence of D-serine (100 µM). NMDAR contribution to _UV_EPSCs was isolated with tetrodotoxin (1 µM), picrotoxin (100 µM), and NBQX (10 µM) for the duration of each experiment. **b** Representative electrophysiological recording of whole-cell _UV_EPSCs in a PV+ interneuron at voltages from −80 to 40 mV (20 mV intervals). Violet bar indicates the time of ultraviolet illumination. **c**_UV_EPSCs in PV+ interneurons exhibit a current-voltage relationship typical of NMDAR-mediated ESPCs (average from *n* = 5 neurons). **d** Representative electrophysiological recording of _UV_EPSCs in a PV+ interneuron voltage-clamped at −55 mV before and during inhibition of NMDARs with 50 µM D-AP5. **e** Time course of normalized _UV_EPSC amplitude in PV+ interneurons (average from *n* = 6 neurons). **f**, **g** D-AP5 reduces (**f**) charge (baseline: −10,691 ± 2113 fC; AP5: −1,777 ± 301.7 fC) and **g** amplitude (baseline: −59.47 ± 7.8 pA; AP5: −10.46 ± 0.71 pA) of _UV_EPSCs in PV+ interneurons (*n* = 6 neurons; sum of signed ranks = 21; *p* = 0.031 for both). Group data (**c**, **e**, **f**, **g**) represented as mean ± SEM. *p < 0.05; Wilcoxon signed ranks test for paired comparisons (**f**, **g**).

Next, we targeted fluorescently labeled PV+ interneurons in acute slices of adult mouse mPFC from PV-Cre::TdTomato mice [69, 70] to test for functional NMDARs electrophysiologically. Given that NMDARs accumulate synaptically and extrasynaptically, we began by uncaging glutamate (MNI-Glu; bath applied) with ultraviolet (UV) light delivered through the microscope objective. With AMPARs, GABA-ARs and voltage-gated sodium channels inhibited (Fig. 2a), we analyzed the current–voltage relationship of excitatory post-synaptic currents (_UV_EPSCs) at a series voltages (−80 to 40 mV, 20 mV intervals; Fig. 2b, c). The current-voltage relationship of _UV_ EPSCs was typical of NMDAR-mediated currents in the presence of extracellular Mg^2+^ [71, 72], and _UV_EPSCs were blocked by the NMDAR antagonist D-AP5 (−55 mV holding potential; Fig. 2d, e). _UV_EPSC charge (Fig. 2f) and peak amplitude (Fig. 2g) were reduced by D-AP5, confirming that _UV_EPSCs represent NMDAR currents.

### All PV+ interneurons contain functional somatodendritic NMDARs

**Fig. 3:**
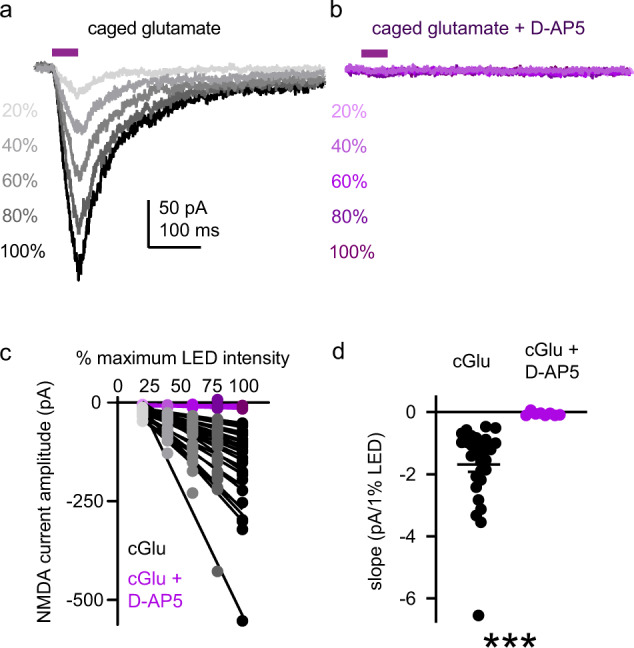
All PV+ interneurons contain functional somatodendritic NMDARs. **a**, **b** Dose-response relationship between LED intensity (20–100% maximum intensity; intervals of 20%) and NMDAR-mediated _UV_EPSC amplitude in representative PV+ interneurons (−55 mV holding potential) in the absence (**a**) or presence (**b**) of D-AP5 (50 µM). **c** Dose-response relationship for each PV+ interneuron tested in the absence (gray; *n* = 29 neurons) or presence (purple; *n* = 8 neurons) of D-AP5. Points represent _UV_EPSC amplitude at a given stimulus intensity for an individual PV+ interneuron. Lines represent the best fit of the dose-response measurement for each neuron determined by simple linear regression. **d** Summary of regression line slopes plotted in **c**. Slopes are steeper for PV+ interneurons in the absence (black; *n* = 29 neurons; −1.69 ± 0.24 pA/1% LED) compared to the presence of D-AP5 (purple; *n* = 8 neurons; −0.06 ± 0.02 pA/1% LED; *U* = 0; *p* < 0.001). Data represented as mean ± SEM. ***p < 0.001; Mann–Whitney test for unpaired comparisons (**d**).

Despite decades of interest and controversy surrounding NMDAR function in PV+ interneurons, the proportion of these neurons with functional NMDARs in adult PFC has not been formally tested. Reports that NMDAR currents are undetectable in some PV+ interneurons are based on synaptic stimulation [32, 58], which is likely to underestimate the number of PV+ interneurons with functional NMDARs. To generate a systematic, unbiased estimate of the proportion of adult mPFC PV+ interneurons with functional NMDARs, we recorded _UV_EPSCs from 37 PV+ interneurons (8 with and 29 without D-AP5; −55 mV holding potential) located 300–850 µm from the pial surface using the approach described above (Fig. 2a). LED intensity was increased in increments of 20% from 20 to 100% in the absence (Fig. 3a) or presence (Fig. 3b) of D-AP5. Using a linear regression, the dose-response relationship between LED intensity and single-trial _UV_EPSC amplitude was quantified for each neuron (Fig. 3c). We then tested whether the slope of this relationship deviated statistically from zero, as predicted for neurons with functional somatodendritic NMDARs. We found that 100% of PV+ interneurons (29 of 29) exhibited NMDAR-mediated _UV_EPSCs, whereas in presence of D-AP5, the slope of the linear regression did not deviate from zero for 37.5% of PV+ interneurons. Moreover, across all neurons, the slope of the regression line was steeper in the absence of D-AP5 than in its presence (Fig. 3d). Together, our data indicate that all PV+ interneurons contain functional somatodendritic NMDARs.

### NMDARs contribute to contralateral prefrontal cortex-evoked synaptic currents in PV+ interneurons

**Fig. 4:**
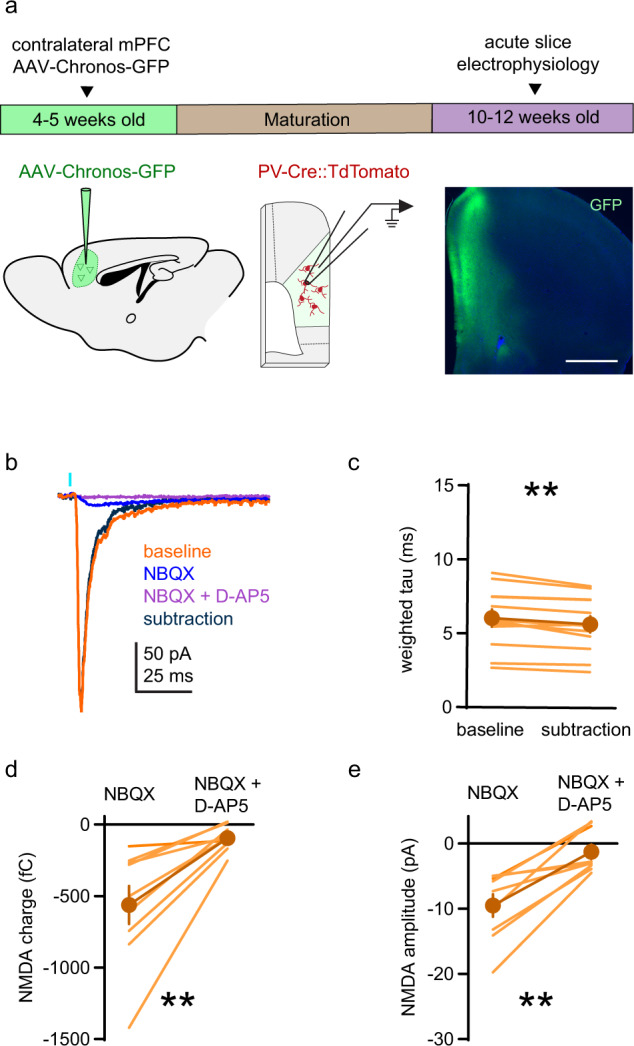
NMDARs contribute to contralateral mPFC-evoked synaptic currents in PV+ interneurons. **a** Timeline and approach used to evoke synaptic currents at contralateral mPFC synapses onto PV+ interneurons (−55 mV holding potential). *Left*: Unilateral microinjection of AAV-Syn-Chronos-GFP into mPFC. *Middle:* TdTomato-labeled PV+ interneurons in the contralateral hemisphere are targeted for recording and EPSCs are evoked optogenetically. *Right*: Coronal section of mPFC showing the injection site of AAV-Syn-Chronos-GFP (green) with DAPI (blue). Scale bar indicates 1 mm. **b** Representative traces of monosynaptic contralateral mPFC-evoked EPSCs in a PV+ interneuron. *Orange*: Baseline EPSC with TTX (1 µM), 4-AP (100 µM), and picrotoxin (100 µM). *Blue*: NMDAR-mediated current measured in the presence of NBQX (10 µM). *Purple*: residual current with NBQX and D-AP5 (50 µM). *Black*: AMPAR-mediated current obtained by digital subtraction of the NMDAR current from the baseline EPSC. **c** EPSC decay (weighted tau) is longer for baseline EPSCs than AMPAR-only EPSCs (subtraction; *n* = 12 neurons; baseline: 6.04 ± 0.59 ms; AMPAR-only; 5.67 ± 0.55 ms; sum of signed ranks = −70; *p* = 0.003). **d**, **e** NMDAR EPSC (**d**) charge (NBQX: −562.7 ± 133.3 fC; NBQX + D-AP5: −94.77 ± 28.85 fC) and **e** amplitude (NBQX: −9.52 ± 1.73 pA; NBQX + D-AP5 −1.28 ± 1.1 pA) are reduced by application of D-AP5 (*n* = 9 neurons; sum of signed ranks = 45; *p* = 0.004 for both). Group data (**c**–**e**) represented as mean ± SEM. **p < 0.01; Wilcoxon signed ranks test for paired comparisons (**c**–**e**).

Having established that all adult mPFC PV+ interneurons express functional NMDARs, we sought to understand the contribution of NMDARs to glutamatergic synaptic currents, including differences between distinct glutamatergic inputs onto these neurons. The latter point is important because pathway-specific contributions of AMPARs and NMDARs have been observed in hippocampal and visual cortical (V1) PV+ interneurons [73–75], which could explain why some studies report little or no synaptic NMDAR current [32, 33, 58]. Since contralateral mPFC is the largest source of long-range glutamatergic input to mPFC PV+ interneurons [76], we initially transduced neurons in the opposite cortical hemisphere with an adeno-associated virus (AAV) coding for the rapidly acting channelrhodopsin Chronos tagged with GFP [77] to measure light-evoked PV+ interneuron EPSCs (see Fig. 4a). Monosynaptic EPSCs (−55 mV holding potential) were isolated by optically stimulating fibers in the presence of TTX, 4-AP and picrotoxin [62, 63] (Fig. 4b). After recording baseline EPSCs, the AMPAR antagonist NBQX was applied to isolate the NMDAR-mediated EPSC component (Supplementary Fig. 2). In a subset of neurons (9 of 12), AP5 was subsequently co-applied with NBQX to test whether the residual current was mediated by NMDARs. Since NMDAR-mediated EPSCs have a longer decay time constant (tau) than AMPAR-mediated EPSCs, we digitally subtracted the NMDAR-mediated current from the baseline current and compared tau of the baseline EPSC and the AMPAR-only EPSC (subtraction; Fig. 4c). Consistent with the presence of NMDARs at contralateral mPFC inputs to PV+ interneurons, tau was longer for baseline EPSCs than for AMPAR-only EPSCs. Moreover, with AMPAR inhibited by NBQX, application of D-AP5 further reduced EPSC charge (Fig. 4d) and amplitude (Fig. 4e), confirming that NMDARs in PV+ interneurons provide a small, yet detectable, contribution to EPSCs originating from contralateral mPFC glutamatergic inputs.

### Larger NMDAR contribution to ipsilateral thalamic compared to contralateral mPFC synaptic currents in PV+ interneurons

**Fig. 5:**
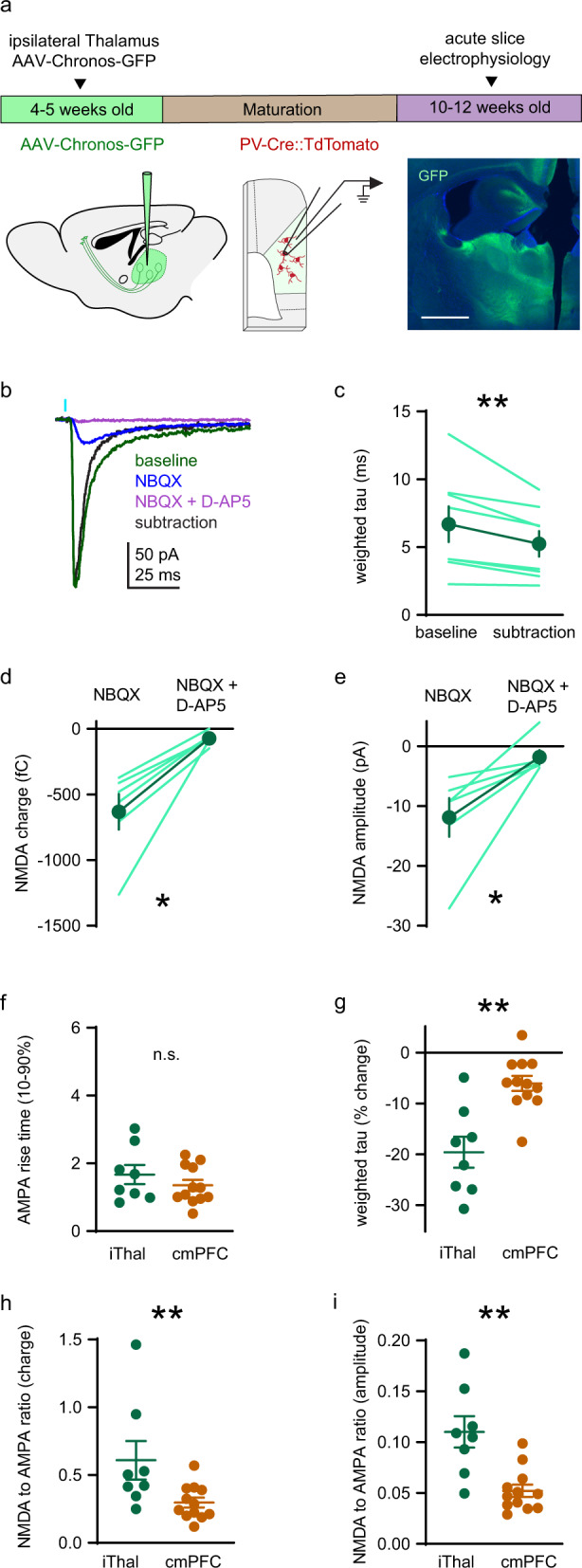
Larger NMDAR contribution to ipsilateral thalamic compared to contralateral mPFC synaptic currents in PV+ interneurons. **a** Timeline and approach used to evoke synaptic currents at ipsilateral thalamic synapses onto PV+ interneurons (−55 mV holding potential), as in Fig. 4 except AAV-Syn-Chronos-GFP injection is targeted to the medial dorsal region of the thalamus. *Right*: Coronal section of the thalamus showing the injection site of AAV-Syn-Chronos-GFP (green) with DAPI (blue). Scale bar indicates 1 mm. **b** Representative traces of monosynaptic ipsilateral thalamus-evoked EPSCs in a PV+ interneuron treated pharmacologically as in Fig. 4. *Green*: Baseline EPSC. *Blue*: NMDAR-mediated EPSC. *Purple*: Residual current with of NBQX and D-AP5. *Black*: AMPAR-mediated current obtained by digital subtraction. **c** EPSC decay (weighted tau) is longer for baseline EPSCs than AMPAR-only EPSCs) (*n* = 8 neurons; baseline: 6.69 ± 1.31 ms; AMPAR-only: 5.24 ± 0.94 ms; sum of signed ranks = −36; *p* = 0.008). **d**, **e** NMDAR EPSC (**d**) charge (NBQX: −632.2 ± 135.1 fC; NBQX + D-AP5: −72.74 ± 19.73) and (**e**) amplitude (NBQX: −11.89 ± 3.23 pA; NBQX + D-AP5 −1.81 ± 1.17 pA) are reduced by application of D-AP5 (*n* = 6 neurons; sum of signed ranks = 21; *p* = 0.031 for both). **f** Rise time (10–90%) of AMPAR-mediated EPSC is similar at ipsilateral thalamus (*n* = 8 neurons; 1.67 ± 0.28 ms) and contralateral mPFC (*n* = 12 neurons; 1.35 ± 0.16 ms) synapses (*U* = 40; *p* = 0.57). **g** Subtraction of the NMDAR-mediated EPSC reduces weighted tau more for ipsilateral thalamus (*n* = 8 neurons; 19.58 ± 3.05%) versus contralateral mPFC (*n* = 12 neurons; 6.04 ± 1.49%) EPSCs (*U* = 10; *p* = 0.002). **h**, **i** NMDA/AMPA ratios by (**h**) charge and (**i**) amplitude are larger at ipsilateral thalamus (*n* = 8 neurons; charge: 0.61 ± 0.14 fC; amplitude: 0.11 ± 0.02 pA) versus contralateral mPFC (*n* = 12 neurons; charge: 0.3 ± 0.04 fC; amplitude: 0.05 ± 0.01 pA) (charge: *U* = 14; *p* = 0.007, amplitude: *U* = 8; *p* = 0.001) synapses. Group data (**c**–**i**) represented as mean ± SEM. *p < 0.05; **p < 0.01; Wilcoxon signed ranks test for paired comparisons (**c**–**e**). Mann–Whitney test for unpaired comparisons (**f**–**i**).

Thalamic innervation of PFC is critical to cognition [78–81]. Therefore, we tested the contribution of NMDARs to ipsilateral thalamus-evoked EPSCs in PV+ interneurons by injecting AAV-Chronos-GFP unilaterally into the medial dorsal region of the thalamus (Fig. 5a). The contribution of NMDARs and AMPARs to monosynaptic EPSCs (−55 mV holding potential; Fig. 5b and Supplementary Fig. 3) was tested as above (Fig. 4b). Tau was longer for baseline EPSCs than for AMPAR-only EPSCs obtained by subtraction (Fig. 5c), indicating that NMDARs contribute to thalamus-evoked EPSCs. Moreover, with AMPARs inhibited, D-AP5 reduced EPSC charge (Fig. 5d) and amplitude (Fig. 5e), confirming that NMDARs contribute to thalamus-derived PV+ interneuron EPSCs.

Our data also suggested that NMDARs contribute relatively more to thalamic EPSCs (Fig. 5c) compared to contralateral PFC EPSCs (Fig. 4c), so we examined this relationship quantitatively. AMPAR EPSCs were kinetically similar at thalamic and contralateral mPFC synapses (Fig. 5f), but NMDARs contribute more to baseline EPSC duration at ipsilateral thalamus compared to contralateral mPFC synapses. Subtracting NMDAR current from baseline EPSCs reduces tau of thalamic EPSCs by 19.58 ± 3.05% compared to 6.04 ± 1.49% for contralateral mPFC EPSCs (Fig. 5g). Furthermore, the ratio of NMDAR to AMPAR (hereafter denoted as NMDA/AMPA ratio) charge (Fig. 5h) or amplitude (Fig. 5i) is approximately twice as large for thalamic EPSCs (charge ratio: 0.61 ± 0.14; amplitude ratio: 0.11 ± 0.02) compared to contralateral mPFC EPSCs (charge ratio: 0.3 ± 0.04; amplitude ratio: 0.05 ± 0.01). Therefore, NMDARs contribute relatively more to excitatory synaptic drive at synapses onto PV+ interneurons that originate from the thalamus. The modest contribution of NMDAR to EPSCS from contralateral mPFC inputs, which are more abundant, may explain why studies that stimulated EPSCs electrically reported little or no synaptic NMDAR current [32, 33, 58].

### NMDARs in PV+ interneurons contribute to thalamus-evoked FFI in mPFC pyramidal neurons

**Fig. 6:**
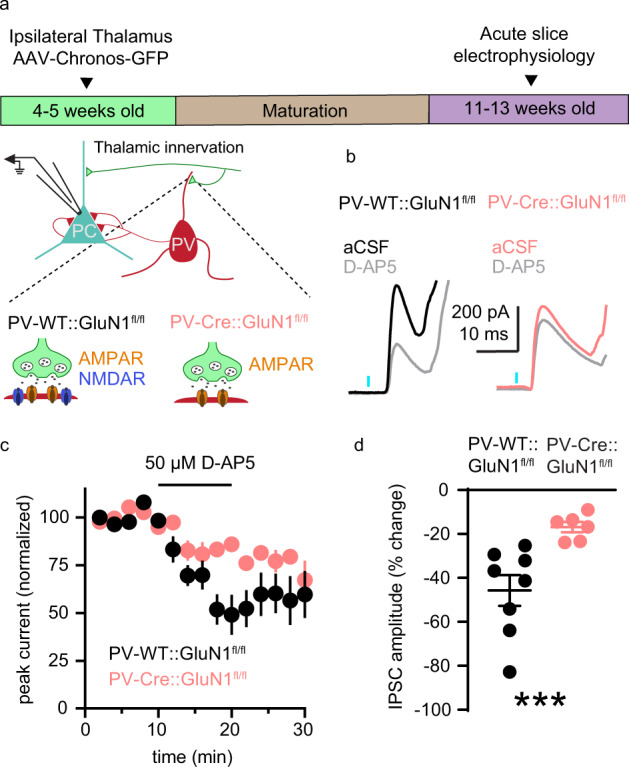
PV+ interneuron NMDAR contribute to thalamus-evoked FFI in mPFC pyramidal neurons. **a** Schematic of the experimental approach used to measure the contribution of PV+ interneuron NMDARs to thalamus-evoked FFI in layer 5/6 pyramidal neurons (0 mV holding potential) in acute coronal slices of adult mPFC. The effect of D-AP5 on IPSC amplitude was compared in mice with GluN1 expression intact (*black*; PV-WT::GluN1^fl/fl^) or GluN1 knocked out (*pink*; PV-Cre::GluN1^fl/fl^) from PV+ interneurons. **b** Representative recordings of feedforward IPSCs in slices from PV-WT::GluN1^fl/fl^ (*left*) or PV-Cre::GluN1^fl/fl^ conditional knockout (*right*) mice in aCSF (*black* or *pink*) and during application of 50 µM D-AP5 (*gray*). **c** Average time course of normalized feedforward IPSC amplitude in PV-WT::GluN1^fl/fl^ (*black*; *n* = 8) or PV-Cre::GluN1^fl/fl^ conditional knockouts (*pink*; *n* = 6) in response to D-AP5 application. **d** D-AP5 decreases feedforward IPSC amplitude more in PV-WT::GluN1^fl/fl^ (*n* = 8 neurons; 45.71 ± 7.0%) than in PV-Cre:: GluN1^fl/fl^ conditional knockouts (*n* = 6 neurons; 16.87 ± 2.37%; *U* = 0; *p* < 0.001). Group data (**c**, **d**) represented as mean ± SEM. ***p < 0.001; Mann–Whitney test for unpaired comparisons (**d**).

Most FFI in thalamocortical circuits is mediated by PV+ interneurons [82, 83] and FFI is important for information processing in cortical circuits [84, 85]. Despite the behavioral and disease relevance of this circuit [78–81, 86], to our knowledge, the contribution of PV+ interneuron NMDARs to FFI of pyramidal neurons in the PFC has never been tested. An obstacle to selectively testing the acute role of NMDARs in PV+ interneurons in PFC function is that bath-applied antagonists will indiscriminately inhibit NMDARs on all neuron types. To overcome this experimental limitation, we compared the effects of D-AP5 application on FFI between Cre-negative GluN1^fl/fl^ mice, which have intact NMDAR expression, and PV-Cre::GluN1^fl/fl^ mice [87] that lack NMDARs selectively in PV+ neurons (Fig. 6a). With the experimenter blind to genotype, we measured the amplitude of feedforward inhibitory post-synaptic currents (IPSCs; 0 mV holding potential) in layer 5/6 pyramidal neurons before and during application of D-AP5 (Fig. 6b, c). We found that in slices from control mice D-AP5 decreased IPSC amplitude by 45.71 ± 7.0%, whereas in slices from PV-Cre::GluN1^fl/fl^ mice the magnitude of the D-AP5 effect was reduced to 16.87 ± 2.37% (Fig. 6d). These findings indicate that the reduction of FFI by D-AP5 in adult mouse PFC is due to inhibition of NMDARs on PV+ interneurons, and not to inhibition of NMDARs on other cell types. Our experiments therefore uncovered an acute role for mature PV+ interneuron NMDARs in shaping thalamo-prefrontal cortical circuit function.

## Discussion

After decades of interest and debate about the expression and function of NMDARs in mature PFC PV+ interneurons, we carried out the first systematic quantitative analysis of the proportion of PV+ interneurons with functional somatodendritic NMDARs in adult PFC (Figs. 1–3). By using fluorescent in situ hybridization and glutamate uncaging, which to our knowledge have not been used previously to address this issue, we found that virtually all PV+ interneurons in the adult rodent mPFC *do* express functional NMDARs, resolving a controversy that has lingered for more than a decade [5, 32–36, 58]. Using pathway-specific optogenetic approaches, we found that NMDARs contribute more to EPSCs in PV+ interneurons at thalamic than contralateral mPFC synapses (Fig. 5). The pathway-specificity of synaptic NMDAR function in PV+ interneurons may explain previous reports that a large percentage of PV+ interneurons do not exhibit synaptic NMDAR currents in adult mPFC, as very small NMDAR currents at synapses from contralateral PFC may have been overlooked in previous studies [32, 58]. This points to a limitation of using electrical stimulation to measure NMDAR EPSCs in mPFC PV+ interneurons. Namely, nonspecific stimulation may be biased to evoke glutamate release from more numerous sources like the contralateral PFC that have limited NMDAR currents, over less numerous ones with more prominent currents like the thalamus. Finally, by combining optogenetic stimulation, PV+ neuron-specific ablation of NMDARs and acute pharmacological manipulation, we discovered that PV+ interneuron NMDARs play a role in thalamus-evoked FFI of PFC pyramidal neurons in adult mice (Fig. 6).

### Implications of NMDAR activity in PV+ interneurons

Together, these experiments represent a significant advance by establishing a defined circuit function for NMDARs in adult mPFC PV+ interneurons, beyond their recognized developmental role [50, 88, 89]. Our observations have major implications for experiments using in vivo manipulations of NMDAR function in humans, primates and rodents [43–47, 49, 51, 55, 90–93] (for review see [54]), and generally support two major hypotheses: (1) that non-competitive NMDAR channel blockers can preferentially inhibit PV+ interneuron NMDAR to produce cortical disinhibition and increase the baseline power of gamma oscillations [31, 54, 94, 95] and (2) that PV+ interneuron NMDAR function contributes to the pathogenesis of psychiatric disorders [20, 96, 97]. For example, these results contextualize the observation that genes associated with psychiatric disorders, like those in the neuregulin/ErbB signaling pathway, regulate NMDAR currents in PV+ interneurons [56, 57], and that NMDAR-relevant markers of oxidative stress in PV+ interneurons and are increased in patients and across models of psychiatric disorders [19, 20, 55, 98]. Furthermore, our findings have implications for the mechanism of action of ketamine as a fast-acting antidepressant, and the possibility that gamma oscillations may be a useful clinical biomarker for the treatment of depression [99, 100]. Finally, the discovery of pathway-specific NMDAR contributions to EPSCs in PFC PV+ interneurons represents a conceptual advance in long-range control of PFC circuit function, suggesting that reduced NMDAR function in PV+ interneurons could have synapse-specific impacts on synaptic integration, long-range connectivity and cognition.

### Implications for thalamo-prefrontal cortex circuit function

Since NMDARs contribute to thalamus-mediated FFI of PFC pyramidal neurons (Fig. 6), it follows that NMDAR hypofunction in PV+ interneurons would alter the temporal relationship between neuronal activity in PFC and upstream brain regions [84] while decreasing the dynamic range of PFC circuits, further degrading function [85]. Thalamic control over the temporal pattern and rate of action potential generation in the PFC is important for cognitive behavior [78, 80, 81], and our work indicates that PV+ interneuron NMDARs are important for this thalamic function. Based on our results, we hypothesize that cognitive functions that require coordinated activity of thalamus and PFC will be particularly impacted by acute, adult inhibition of PV+ interneuron NMDAR but testing this hypothesis will require technical advances. Furthermore, reduced PV+ interneuron-mediated FFI due to NMDAR hypofunction represents a molecular and circuit-based mechanism to explain reduced functional connectivity between higher-order thalamus and PFC in Scz patients, as well as cognitive deficits elicited by non-competitive NMDAR antagonists and observed in psychiatric disorders [3, 38, 86]. Notably, PV+ interneuron-mediated FFI is independent of NMDAR in somatosensory cortex [82], consistent with the observation that there are substantial differences between PV+ interneuron-mediated FFI in PFC and somatosensory cortex, including a slower time course of FFI onset in PFC [83]. Mechanistic differences in PV+ interneuron-mediated FFI may support different computational requirements of PFC and primary sensory cortices.

### Pathway-specific NMDAR function in PV+ interneurons

Our finding that NMDARs contribute more to adult PV+ interneuron EPSCs at thalamo-prefrontal synapses, relative to corticocortical synapses (Figs. 4, 5), points to pathway-specific differences in information processing at glutamatergic inputs that drive PV+ interneuron activity. Therefore, differences in stimulation parameters or brain state might explain conflicting evidence about whether NMDAR antagonists produce disinhibition in the PFC [33, 44, 101, 102]. Input-specific differences have been reported in visual cortical and hippocampal PV+ interneurons [73–75], where EPSPs evoked by glutamate uncaging exhibit NMDAR-dependent supralinear summation in dendrites [75]. Beyond impacting synaptic integration, pathway-specific NMDAR and AMPAR expression patterns enforce differing rules of synaptic plasticity in hippocampal interneurons [73, 103, 104], and may in PFC as well. Furthermore, although we focused on long-range inputs to PV+ interneurons here, PFC PV+ interneurons also receive abundant glutamatergic innervation from local pyramidal neurons [76]. NMDAR currents are stronger at feedback compared to feedforward inputs to hippocampal PV+ interneurons [73], where they may contribute to the formation of stable neural ensembles [75]. Testing the contribution of NMDAR at local recurrent synapses onto PV+ interneurons may offer further insight into reports of synaptic currents devoid of an NMDAR-mediated component and will benefit our understanding of PFC circuit function.

Here we focused on co-expression of *Grin1* and *Grin2b* in adult PFC PV+ interneurons to establish the proportion of neurons expressing the minimal transcripts necessary to produce functional NMDAR. Given that GluN2 subunit composition changes across development and confers NMDAR with functional diversity [66], in future studies it will be informative to test whether PV+ interneuron NMDARs with distinct subunit composition contribute to discreet cellular or circuit function in adult PFC, and whether developmental changes in subunit composition play a causal role in establishing adolescence as a sensitive period for pathway-specific maturation of GABAergic neurotransmission in PFC [105]. Taken together, recent studies which indicate that a GluN2C/2D-specific positive allosteric modulator impacts both excitability and NMDAR-mediated EPSCs in adult PFC fast-spiking interneurons [106], that mice lacking the GluN2A subunit in PV+ interneurons exhibit a blunted electrophysiological response to ketamine in V1 [107], and that PV+ interneurons can exhibit pathway-specific changes in NMDA/AMPA ratio across developmental critical periods [74] underscore the point that nuanced, input-specific analysis will be required to understand the contribution of distinct GluN2 subunits in PV+ interneurons to adolescent PFC maturation and adult function. Despite the challenges, further understanding of pathway-specific mechanisms of glutamatergic transmission in PFC PV+ interneurons during development and adulthood is critical to advance the field’s understanding of PFC function, and may contribute to explanations of cognitive impairment in developmental psychiatric disorders like Scz and ASD.

Our results partially contrast with the only other study to compare NMDA/AMPA ratios at long-range inputs to PFC PV+ interneurons [36]. No significant difference in NMDA/AMPA ratios was reported between thalamic, ventral hippocampal, and contralateral PFC inputs to PV+ interneurons, although there was a trend toward greater NMDAR contribution at thalamic inputs compared with the other two [36]. This discrepancy is likely the result of two main differences in experimental approach. First, we used Cs+-based, rather than K+-based, internal solution to enhance space clamp therefore improving our ability to resolve currents throughout the dendritic arbor. Second, we used a combination of TTX and 4AP to pharmacologically isolate monosynaptic inputs, as suggested previously [62], prior to measuring NMDA/AMPA ratios to reduce measurement errors stemming from polysynaptic EPSCs.

### Significance of NMDAR function in PV+ interneurons for high-frequency cortical activity

Since PV+ interneurons exhibit functional specializations to respond to glutamatergic input with high temporal fidelity [8], including expression of Ca^2+^-permeable AMPAR with rapid kinetics [58, 82, 108], an influential perspective has held that the comparatively long EPSCs produced by NMDARs are at odds with generation of temporally precise action potentials and gamma oscillations [5, 34, 35]. Since virtually all PV+ interneurons contain NMDARs (Figs. 1–3), high-frequency temporal fidelity *must* be compatible with prolonged somatodendritic excitatory currents generated by NMDARs. In contrast to the comparatively quiescent conditions in acute brain slices, in vivo, fast AMPAR-mediated events may be superimposed on slower NMDAR currents, thereby increasing the probability that AMPAR-mediated events induce action potentials. This hypothesis is supported by data indicating that, during periods of elevated cortical activity, PV+ interneurons exhibit prolonged depolarizations [109, 110] to voltages at which PV+ interneuron NMDAR Mg^2+^ block is substantially reduced (Fig. 2) and is also consistent with recent work suggesting that extrasynaptic NMDARs contribute to GABAergic interneuron excitability [111]. Furthermore, like NMDAR antagonists, PV+ neuron-specific knockout of NMDAR is detrimental to cognitive function and increases baseline gamma power [54]. Considered alongside our data, this indicates that NMDAR currents in PV+ interneurons contribute to healthy PFC function, whether or not PV+ interneurons could theoretically generate more temporal precision or higher power gamma oscillations without them.

### Summary

By demonstrating that nearly all PV+ interneurons express functional NMDARs, and that their contribution to EPSCs is pathway-specific, we have resolved a major controversy in the field and provided an explanation for previous reports that most PV+ interneurons in adult PFC do not exhibit synaptic NMDAR currents. Furthermore, our data reveal a defined circuit function for NMDARs in adult PV+ interneurons that has previously eluded the field, and our findings potentially offer a molecular and circuit-based explanation for why cognitive impairments emerge under conditions of reduced NMDAR function in PV+ interneurons. In the future, it will be interesting to test how NMDAR function in PV+ interneurons contributes to connectivity between distal brain regions and PFC, as well as to behaviors that depend on that connectivity. Furthermore, it will be interesting to test the extent to which models of psychiatric disease exhibit altered NMDAR function in PV+ interneurons, how this is related to oxidative stress and whether pharmacological interventions that target NMDARs with distinct subunit composition can ameliorate deficits in PV+ interneuron function.

## Supplementary information

Supplementary Materials

Parvalbumin interneuron NMDA receptors in female and male animals.

NBQX reduces contralateral PFC EPSC size in PV+ interneurons.

NBQX reduces ipsilateral thalamic EPSC size in PV+ interneurons.
