# Supplementary material for: Pathway-specific contribution of parvalbumin interneuron NMDARs to synaptic currents and thalamocortical feedforward inhibition
Source: Mol Psychiatry. 2022 Sep 8;27(12):5124–34. doi: 10.1038/s41380-022-01747-9 (PMC9763122; doi:10.1038/s41380-022-01747-9)
Supplement: Supplementary file 1 — Supplementary Materials [file 41380_2022_1747_MOESM1_ESM.docx]

**Supplementary Figure Legends**

**Figure S1: Parvalbumin interneuron NMDA receptors in female and male animals.**

***a***) *Grin1* is expressed by a similar proportion of adult PFC *Pvalb*+ neurons in female (n = 3; 90 ± 3.9%) and male (n = 3 mice; 96 ± 0.9%) mice (U = 2; p = 0.4). ***b***) *Grin2b* is expressed by a similar proportion of adult PFC *Pvalb*+ neurons in female (n = 3; 95 ± 2%) and male (n = 3 mice; 99 ± 1%) mice (U = 1; p = 0.2). ***c***) LED/NMDAR current relationship is similar between female (n = 13 neurons; -1.41 ± 0.19 pA/1% LED) and male (n = 16 neurons; -1.91 ± 0.4 pA/1% LED) mice (U = 102; p = 0.94). ***d***) peak NMDAR-mediated current amplitude induced by glutamate uncaging is similar between female (n = 13 neurons; -136.9 ± 17.3 pA) and male (n = 16 neurons; 174.8 ± 33.1 pA) mice (U = 104; p > 0.99). Data represented as mean ± SEM. Mann-Whitney test for unpaired comparisons.

**Figure S2: NBQX reduces contralateral PFC EPSC size in PV+ interneurons.**

**a**) EPSC charge is reduced by NBQX (baseline: -2845 ± 649.7 fC; NBQX: 553.1 ± 105.4 fC, n = 12 neurons; sum of signed ranks = 78; p < 0.001). (***b***) EPSC amplitude is reduced by NBQX (baseline: -203.2 ± 36.5 pA; NBQX: -9.2 ± 1.4 pA, n = 12 neurons; sum of signed ranks = 78; p < 0.001). ***p < 0.001; Wilcoxon signed-ranks test for paired comparisons.

**Figure S3: NBQX reduces ipsilateral thalamic EPSC size in PV+ interneurons.**

**a**) EPSC charge is reduced by NBQX (baseline: -2542 ± 537.6 fC; NBQX: -778.8 ± 146.9 fC, n = 8 neurons; sum of signed ranks = 36; p = 0.008). (***b***) EPSC amplitude is reduced by NBQX (baseline: -122.8 ± 18.3 pA; NBQX: -12.5 ± 2.4 pA, n = 8 neurons; sum of signed ranks = 36; p = 0.008). **p < 0.01; Wilcoxon signed-ranks test for paired comparisons.

**Supplementary Methods**

**Animal subjects:** Adult (P70-100) male and female mice were used for all experiments. For *in situ* hybridization experiments, C57BL/6J (JAX #000664) were obtained from Jackson Laboratories (Bar Harbor, ME, USA) at four weeks or bred in-house, and were group housed until the time of the experiment. For electrophysiological experiments, mice were bred in house and weaned at 3 weeks. PV-IRES-Cre (Stock #017320)^69^, ai14 (Cre-dependent TdTomato reporter line; Stock # 007914)^70^ and *Grin1*^fl/fl^ (Stock #005246)^87^ mice were obtained from Jackson Laboratories. To generate PV-Cre::TdTomato mice (**Fig. 2-5**), mice homozygous or heterozygous PV-IRES-Cre were crossed with homozygous ai14 mice. To generate PV-Cre::GluN1^fl/fl^ mice and Cre-negative::GluN1^fl/fl^ littermates to serve as controls (**Fig. 6**) homozygous *Grin1*^fl/fl^ males were crossed with GluN1^fl/fl^ or GluN1^fl/wt^ females that were also heterozygous for PV-IRES-Cre. Mice were genotyped by PCR using the following primers (listed 5’ to 3’): **PV-IRES-Cre:** IRESfc: CTT GGA ATA AGG CCG GTG TG; IRESr2: GCA GAG GCA CCT GTC GCC AG; oIMR8290: CAG AGC AGG CAT GGT GAC TA; oIMR8291: AGT ACC AAG CAG GCA GGA GA (WT: ≈ 500 bp; PV-IRES-Cre: ≈ 250 bp) OR 16210: CCA TTC CCT CAT CCA CAG AG; 27606: GAC GCA ATC GTC CGA TCC; 49521: CAA ACA AAC TGA ACA GAA ACT CAG G (WT: ≈ 280 bp; PV-IRES-Cre: ≈ 690 bp); **ai14:** IMR9020: AAG GGA GCT GCA GTG GAG TA; IMR9021: CCG AAA ATC TGT GGG AAG TC; IMR9103: GGC ATT AAA GCA GCG TAT CC; IMR9105: CTG TTC CTG TAC GGC ATG G (WT: ≈ 300 bp; Mutant: ≈ 200 bp); ***Grin1*^fl/fl^:** 11466: GTG CTG GGA TCC ACA TTC AT; 11467: 5’- AAA CAG GGC TCA GTG GGT AA (WT: ≈ 230 bp; *Grin1*^fl^: ≈ 400 bp).

**Fluorescent *In situ* hybridization:** *Tissue processing.* Fluorescent in-situ hybridization for *Pvalb*, *Grin1*, and *Grin2b* was performed as previously described, using RNAscope Fluorescent Multiplex Assay (Advanced Cell Diagnostics (ACD); Newark, CA, USA)^60^. Fresh-frozen mPFC tissue sections were obtained after decapitation of deeply anesthetized mice (isofluorane). Briefly, brains were quickly removed and a block of tissue containing mPFC was cut, flash-frozen by submersion in liquid nitrogen (≈ 15 s) and stored at -80°C. Prior to cryosectioning (12 μm section thickness on a Leica CM3050 S Cryostat; Leica Biosystems; Deer Park, IL, USA), brains were equilibrated overnight at -20°C to prevent tissue cracking during cryosectioning. Sections were subsequently stored on slides at -80°C. Immediately prior to *in situ* hybridization, mPFC sections were fixed on the slides by immersion in chilled 4% PFA for 15 minutes. Sections were washed twice in 1x PBS for 2 min, dehydrated in increasing concentrations of ethanol for 5 min (50%, 70%, and 100% x 2) and dried on the bench. A hydrophobic barrier was drawn around sections before incubation in Protease IV solution at room temperature for 30 min. Sections were rinsed twice in 1x PBS before proceeding with the RNAscope Multiplex Assay.

The RNAscope Multiplex Assay was carried out as described previously^60^. Probes were obtained from ACD (*Pvalb*: Cat # 407821-C2; *Grin1*: Cat # 431611; *Grin2b*: Cat # 417391-C3) and incubations took place in a humidifying chamber at 40°C with two 5 min washes in RNAscope Wash Buffer (ACD) at room temperature between incubation periods. Three drops of amplification solutions were used for sequential amplification incubations of 30 min (AMP1), 15 min (AMP2), and 30 min (AMP3) min. Three drops AMP4A solution (15 min incubation) was used to label Ch1 probes (*Grin1*) with Alexa488, Ch2 probes (*Pvalb*) with Atto550, and Ch3 probes (*Grin2b*) with Atto647. Finally, nuclei were labeled by 30 sec DAPI application. Slides were coverslipped using DABCO mounting media and stored at 4°C until image acquisition.

*Image acquisition.* Z-stacked images including mPFC were acquired using a Zeiss LSM 780 (Carl Zeiss Microscopy; White Plains, NY, USA) microscope with a 20x objective at 2x zoom. This approach produced a large (≈ 3 x 1.5mm) tiled image which allowed us to analyze the entirety of prelimbic (PrL) and infralimbic (ILA) cortex as defined by the Allen Brain Atlas. This region will hereafter be referred to as mPFC. Image acquisition settings were set based on a representative section of mPFC and kept constant across all samples.

*Image analysis.* Images were stitched in Zen and preprocessed in ImageJ/Fiji (www.imagej.net) before automated analysis in CellProfiler 4 (www.cellprofiler.org; Broad Institute; Cambridge, MA, USA)^64^. For preprocessing, image stacks were imported to ImageJ/Fiji under a split channel view. Channels were merged then rotated until the midline was vertical. The stack was converted to a maximum intensity projection and the polygon selection tool was used to define the mPFC for subsequent analysis. The selection area was converted into a mask to exclude brain areas outside of mPFC from analysis. Images were split into separate channels, converted to 8-bit grayscale images, and saved as separate TIF files.

For analysis, preprocessed TIF files for each channel for each subject were sequentially run through an automated CellProfiler pipeline that was kept constant across all samples. Briefly, a white top-hat filter was used to enhance fluorescent mRNA signal in each channel. The enhanced signal was used to identify primary RNAscope signal for each channel by adaptive (*Grin1* and *Grin2b*) or global (*Pvalb*) otsu thresholding strategies. Cells were identified based on the presence of DAPI, where a defined 14 μm cell diameter was produced by nine pixel expansion from the center of identified DAPI signal. Cells were then related to the primary RNAscope signal and defined as positive for each mRNA of interest based on a minimum number of fluorescent puncta (2 puncta for *Pvalb* and 3 puncta for *Grin1* and *Grin2b* each). Cell location relative to midline was calculated by CellProfiler based on the distance from the midline (left edge of the cropped image) to the centroid of the identified cell. Laminar designations were made according to Anastasiades and colleagues, 2019^68^. Data was exported to CSV files for quantification and statistical analysis. One sample was excluded from analysis for background signal that interfered with automated cell detection.

**Electrophysiology:** *Slice preparation.* Slices were prepared using the N-methyl-D-glucamine (NMDG) protective recovery method as described previously^112^. Prior to preparation of brain slices for electrophysiological recordings, adult mice (P70 - 100) were deeply anesthetized with i.p. Avertin and transcardially perfused with 25 ml oxygenated (95% O_2_, 5% CO_2_) ice-cold NMDG artificial cerebrospinal fluid (aCSF) solution (pH: 7.3 – 7.4; 300 – 310 mOsmol/kg) containing the following (in mM): NMDG 92; HEPES 20; Glucose 25; NaHCO_3_ 30; NaH_2_PO_4_ 1.2; KCl 2.5; Na-ascorbate 5; Na-pyruvate 3; Thiourea 2; MgSO_4_ 10; CaCl_2_ 0.5. Following decapitation, brains were quickly removed and 300 µm coronal slices containing mPFC were cut in ice-cold NMDG-aCSF using a Leica VT1200S vibratome. After cutting was complete, slices were transferred to warm ≈ 34°C, continuously oxygenated (95% O_2_, 5% CO_2_) NMDG-aCSF. During the first 20 min of incubation, Na+ concentration in NMDG-aCSF was progressively increased according to the protocol for 1 – 3 month old mice published by Ting and colleagues^112^. After 25 min in NMDG-aCSF, slices were transferred to room temperature (RT) holding aCSF (aCSFh) containing the following (in mM): NaCl 92; HEPES 20; Glucose 25; NaHCO_3_ 30; NaH_2_PO_4_ 1.2; KCl 2.5; Na-ascorbate 5; Na-pyruvate 3; Thiourea 2; MgSO_4_ 1; CaCl_2_ 2. Slices were kept in aCSFh (continuously oxygenated; pH: 7.3 – 7.4; 300 – 310 mOsmol/kg) for at least 1 hr prior to electrophysiological recording. Recording aCSF (aCSFr; pH: 7.3 – 7.4; 300 – 310 mOsmol/kg) containing (in mM): NaCl 125; NaH_2_PO_4_ 1.2; KCl 2.5; NaHCO_3_ 26; Glucose 11; MgCl 1.3; CaCl_2_ 2.5, was continuously oxygenated and delivered to the recording chamber with a pump at ≈ 2 ml/min. Experiments were conducted at 30-32°C.

*Data acquisition.* Whole-cell recording pipettes (2-4 MΩ) were pulled from borosilicate glass capillaries (1.5 mm outer diameter, 1.12 mm inner diameter; World Precision Instruments; Sarasota, FL, USA) using a Flaming-Brown horizontal puller (P97; Sutter Instruments; Novato, CA, USA). Recording pipettes were filled with freshly filtered (0.22 µm syringe filter) internal solution (pH: 7.3 – 7.4 adjusted with CsOH; ≈ 305 mOsmol/kg) containing the following (in mM): CsMeSO_3_ 115; CsCl 20; HEPES 10; EGTA 0.6; Spermine 0.1; MgCl 2.5; Na-Phosphocreatine 10; Na-ATP 4; Na-GTP 0.3; QX-315 Chloride 1. For recordings of feedforward inhibition in pyramidal neurons, 1 mM MK-801 (Tocris; Minneapolis, MN, USA) was added to the internal solution to selectively inhibit NMDAR in the neuron being recorded. PV+ interneurons in PrL and dorsal IL (Allen Brain Atlas; ≈ 270 – 850 µm from midline) were targeted for recording based on TdTomato expression in PV-Cre::TdTomato mice, and pyramidal neurons in PrL and dorsal IL (Allen Brain Atlas; ≈ 475 – 800 µm from midline) were targeted based on morphology. TdTomato was excited for visualization using illumination from a CoolLED pE-300^ultra^ (CoolLED; Andover, UK) delivered to the slice through a 40x Nikon (Tokyo, Japan) water immersion objective. Fluorescent and infrared differential interference contrast images were detected using a Hamamatsu ORCA-Flash4.0LT camera (Hamamatsu; Hamamatsu City, Japan), and visualized using Nikon Elements software. Images of the of the recording pipette within mPFC for post hoc estimation of recording location relative to midline were captured using the same camera and software using a 4x Nikon objective. Whole-cell voltage-clamp recordings were conducted using a computer-controlled amplifier (Multiclamp 700B; Molecular Devices; San Jose, CA, USA), digitized (Digidata 1550B; Molecular Devices), and acquired with Clampex 11 (Molecular Devices). Recordings were acquired at a sampling rate of 20 kHz with a low-pass filter set at 10 kHz. Access resistance (Ra) was monitored for the duration of each experiment, and data was excluded if Ra changed more than 25% or exceeded 25 MΩ. The liquid junction potential (7 mV) was corrected^113^.

*Glutamate uncaging.* MNI-Glutamate (MNI-Glu; 50 µM; Tocris) was recirculated through the bath for the duration of each experiment. To test for the presence functional NMDAR in PV+ interneurons, 2,3-Dioxo-6-nitro-1,2,3,4-tetrahydrobenzo[f]quinoxaline-7-sulfonamide (NBQX; 10 µM; Tocris), picrotoxin (100 µM; Tocris), and tetrodotoxin (TTX; 1 µM; Tocris; were always co-applied with MNI-Glu inhibit AMPAR, GABA-AR, and action potential-dependent neurotransmission, respectively. Since NMDAR activation requires co-agonist binding, D-serine (100 µM; Tocris) was also applied for the duration of these experiments. For experiments which included application of D-AP5 (50 µM; Tocris), NBQX, picrotoxin, TTX, and D-serine were applied as well. To uncage glutamate on target PV+ interneurons, 50 ms pulses (one pulse every 30 s) of ultraviolet (UV) light were triggered by a digital output from the Digidata 1550B, generated by a CoolLED pE-300^ultra^, and delivered to the slice through a 40x objective focused on the cell body of the PV+ interneuron being recorded.

*Synaptic stimulation.* To optogenetically stimulate contralateral mPFC and ipsilateral thalamic terminals in mPFC, blue light pulses (1 ms every 10 s for EPSCs in PV+ interneurons; 0.5 ms every 20 – 30 s for feedforward inhibition in pyramidal neurons) were triggered by a digital output from the Digidata 1550B, generated using a CoolLED pE-300^ultra^, and delivered to the slice through a 40x objective focused on the cell body of the neuron being recorded. To isolate monosynaptic EPSCs in PV+ interneurons, aCSFr was modified to contain 1 µM TTX to prevent action potential-mediated synaptic transmission, along with 4-aminopyridine (4-AP; 100 µM; Sigma Aldrich; St. Louis, MO, USA) and 4 mM Ca^2+^ to facilitate Chronos-mediated neurotransmitter release^62, 63^. To measure the contribution of NMDAR EPSCs at subthreshold voltages, PV+ interneurons were voltage clamped at -55 mV with 100 µM picrotoxin in the bath to inhibit GABA-AR for the duration of each experiment. After a baseline period, 10 µM NBQX was bath applied for at least 5 min to inhibit AMPAR and test for an NMDAR-mediated component of the EPSC. In a subset of recordings, 50 µM D-AP5 was subsequently coapplied with 10 µM NBQX to test whether residual current in the presence of NBQX was mediated by NMDAR.

The experimenter was blind to genotype for IPSC recordings in pyramidal neurons, and standard aCSFr (described above) was used to record feedforward IPSCs. To isolate feedforward IPSCs, pyramidal cells were voltage clamped at 0 mV, the approximate reversal potential of ionotropic glutamate receptor-mediated currents. Furthermore, 1 mM MK-801 was added to the internal solution for these experiments to inhibit NMDAR-mediated currents in the neuron being recorded^61^. This also served to minimize the direct impact of D-AP5 application on the pyramidal cell in which feedforward inhibition was being measured. The effect of D-AP5 (50 µM; 10 min application) was tested in neurons which exhibited a reliable IPSC peak within 10 ms of the onset of optogenetic stimulation. The time-to-peak criteria was based on the characterization of PV+ interneuron-mediated thalamocortical FFI in a similar region of mouse PFC that was conducted by Delevich and colleagues in 2015^83^. Compared to PV+ interneuron-mediated thalamocortical FFI in sensory cortices, EPSC to IPSC delay is substantially longer in mPFC pyramidal neurons, with mean IPSC onset ≈ 10.2 ms after optogenetic stimulation^83^. Optogenetic stimulation intensity was adjusted so that the first IPSC peak was approximately half of the maximum amplitude observed.

*Electrophysiological data analysis.* Electrophysiological data was analyzed offline using Clampfit. The experimenter was blind to genotype for analysis of data comparing mice of different genotypes (**Fig. 6**). Data testing the dose-response relationship between LED intensity and _UV_EPSC amplitude was low-pass filtered at 1 kHz and all other data was filtered at 2 kHz prior to further analysis in Clampfit. Other than peak amplitude measurements for the _UV_EPSC LED-response relationship which were based on single trials at each stimulation intensity, all other analysis for statistical comparisons was conducted on traces averaged across multiple trials (_UV_EPSC current/voltage curve: 6 trials; _UV_EPSC D-AP5 sensitivity: 4 trials; PV+ interneuron EPSCs: 12 trials; feedforward IPSCs: 10 – 15 trials). Representative traces in figures are averages of the same number of trials used for statistical comparisons. To construct time courses depicting normalized current amplitude, peak amplitude was measured for each trial and either directly normalized based on the mean peak amplitude (_UV_EPSCs) or averaged in 2 min bins and then normalized to mean peak amplitude during the baseline period (feedforward IPSCs).

Measurements of peak amplitude and charge transfer were made relative to a 200 ms period immediately preceding glutamate uncaging or synaptic stimulation. _UV_EPSC charge transfer was measured over the course of 1 s beginning at the onset of the uncaging stimulus, and peak amplitude was defined as the maximal deviation from baseline to occur within 60 ms of the onset (10 ms after termination) of the uncaging stimulus. For optogenetically evoked EPSCs in PV+ interneurons, charge transfer was measured over the course of 200 ms beginning at the onset of the optogenetic stimulus, and peak amplitude was defined as the maximal deviation from baseline to occur within the same time window. To analyze the AMPAR mediated component of optogenetically evoked EPSCs, the NMDAR mediated current (obtained in the presence of NBQX) was digitally subtracted from the baseline current. NMDA/AMPA ratios were calculated by dividing the peak amplitude or charge carried by the NMDAR-mediated current by the same measurements obtained from the digital subtraction. To test whether NMDAR-mediated currents prolong the duration of PV+ interneuron EPSCs, the decay phase of the baseline current (≈ 90% - 10% peak amplitude) was fit with a double exponential curve using the Levenberg-Marquardt method. Next, the same time points were used to fit the AMPAR-mediated current. Weighted tau for baseline and AMPAR currents were calculated as $\frac{(\left( A_{1}\times\tau_{1} \right)+\left( A_{2}\times\tau_{2} \right))}{\left( A_{1}+A_{2} \right)}$. Percent change in tau was calculated as $(\frac{\tau_{AMPA-}\tau_{baseline}}{\tau_{baseline}})\times100$. Peak amplitude of feedforward IPSCs in pyramidal neurons was measured using the first outward-going peak after stimulus onset. Percent change in the feedforward IPSC was calculated as $(\frac{{Peak}_{AP5-}{Peak}_{aCSF}}{{Peak}_{aCSF}})\times100$.

**Stereotaxic injection:** Stereotaxic injections were conducted under isoflurane anesthesia (3% induction; 1 - 2% maintenance) using a stereotaxic instrument (Kopf). Unilateral injections (200 µl per infusion site at 1 - 5 nl/s) of a viral suspension containing AAV5-Syn-Chronos-GFP (Addgene; Watertown, MA, USA; #59170-AAV5) were targeted to either mPFC (from bregma in mm: AP +1.75; ML ± 0.4; DV -2.9, -2.7, -2.5) or the medial dorsal region of the thalamus (from bregma in mm: AP -1.1; ML ± 0.37; DV -3.6, -3.45, -3.3). The glass capillary used for viral infusion was left in place for at least 5 min following the most dorsal injection before being slowly withdrawn from the brain. Wounds were closed using sutures (DemeTECH; Miami Lakes, FL, USA), and ketoprofen (5 mg/kg i.p.) was administered immediately after the surgery, and again 24 and 48 hr later to provide postoperative analgesia. Injection sites were visualized with native GFP fluorescence and captured using an EVOS M5000 microscope (ThermoFisher; Waltham, MA, USA).

*Statistical analysis.* Statistical analyses were conducted in GraphPad Prism (Version 8.4.3; GraphPad Software; San Deigo, CA, USA). Statistical comparisons between groups were two-tailed and made using the Wilcoxon signed-ranks test for paired comparisons or the Mann-Whitney test for unpaired comparisons. P-values were obtained for individual neurons’ glutamate uncaging LED-response relationships using a simple linear regression. A linear regression was used instead of a saturating function based on our empirical observation that response amplitude was not saturating under our conditions. Sample size was based on established practices in the field. Group assignment for animal subjects was not systematically randomized. In all cases p < 0.05 was used as the threshold for significance. Data are represented as mean ± SEM.
