# Supplementary figures and images for: Pathway-specific contribution of parvalbumin interneuron NMDARs to synaptic currents and thalamocortical feedforward inhibition
Source: Mol Psychiatry. 2022 Sep 8;27(12):5124–34. doi: 10.1038/s41380-022-01747-9 (PMC9763122; doi:10.1038/s41380-022-01747-9)

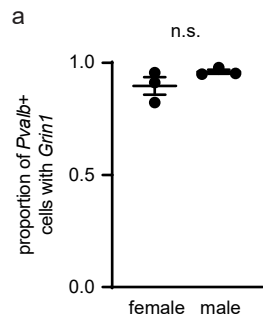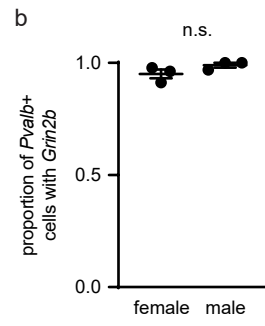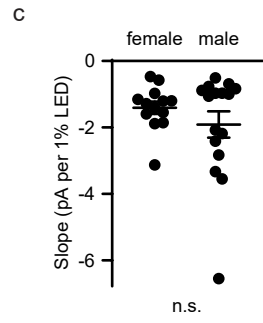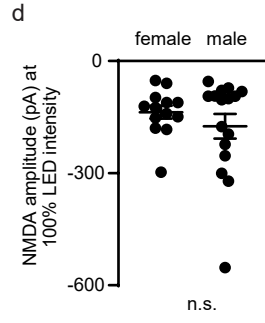

Supplement: Supplementary file 2 — Parvalbumin interneuron NMDA receptors in female and male animals. [file 41380_2022_1747_MOESM2_ESM.pdf]

a

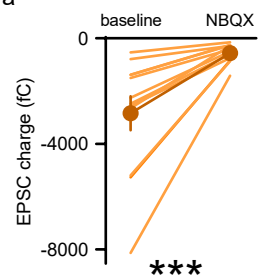

b

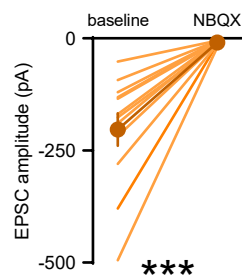

Supplement: Supplementary file 3 — NBQX reduces contralateral PFC EPSC size in PV+ interneurons. [file 41380_2022_1747_MOESM3_ESM.pdf]

a

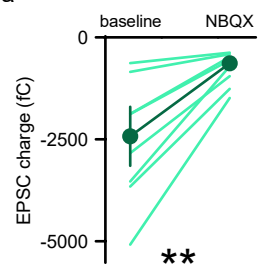

b

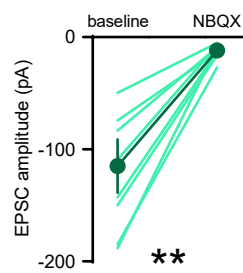

Supplement: Supplementary file 4 — NBQX reduces ipsilateral thalamic EPSC size in PV+ interneurons. [file 41380_2022_1747_MOESM4_ESM.pdf]
